# Social Media Use and Health: What We Have Known So Far, and What We Need to Investigate in the Future

**DOI:** 10.3390/healthcare14172771

**Published:** 2026-09-01

**Authors:** Shaoyu Ye, Kevin K. W. Ho

**Affiliations:** 1 Institute of Library, Information and Media Science, University of Tsukuba, Ibaraki 305-8550, Japan; 2 Institute of Business Sciences, University of Tsukuba, Tokyo 112-0012, Japan; ho.kevin.ge@u.tsukuba.ac.jp

**Keywords:** social media use, physical health, mental health, social health, misinformation, health policy

## Abstract

This review examines the evolving relationship between social media use and health from the perspective of the World Health Organization (WHO), which defines health as a state of complete physical, mental, and social well-being. Drawing on recent empirical findings and systematic reviews, this paper synthesizes current evidence regarding how social media use relates to the three dimensions of health and may facilitate the spread of health misinformation. As social media has become deeply integrated into daily life, understanding these dynamics is increasingly important for developing evidence-based strategies that promote public health in the digital era. Under the theme “What We Have Known so far,” this review highlights the dual nature of social media as a double-edged sword. On one hand, social media facilitates health promotion, knowledge sharing, social support, and the development of social capital and interpersonal relationships, improving the three dimensions of well-being. On the other hand, excessive or problematic use has been associated with adverse mental, physical and social health outcomes, while exposure to online misinformation poses substantial challenges to effective public health communication. This review further emphasizes that social media impacts vary across platforms, user characteristics, patterns of engagement, and combinations of platforms. Under the theme “What We Need to Investigate in the Future,” several research priorities are identified. These include adopting an integrated framework that simultaneously considers physical, mental, and social health; moving beyond cross-sectional designs toward longitudinal and causal approaches and examining platform-specific effects and the influence of individuals’ broader social media portfolios; incorporating objective behavioral measures alongside self-reported data and investigating the emerging role of generative artificial intelligence in health communication and misinformation; and developing evidence-based interventions to improve social media literacy and promote healthier patterns of use. Overall, this review argues that future research should move beyond asking whether social media is beneficial or harmful to health and instead identify when, how, for whom, and under what circumstances it promotes or compromises well-being. Such an integrated perspective will provide a stronger scientific foundation for public health policies and healthier digital societies.

## 1. Introduction

Social media has become deeply integrated into nearly every aspect of our lives. People use different social media platforms to maintain connections with their friends and relatives, access and share information, and collaboratively create content with others. Consequently, social media use is closely intertwined with everyday routines and social interactions. However, prior research has yielded mixed findings regarding its relationships with health. Some studies suggest that social media use can be beneficial, whereas others indicate potential adverse consequences. For example, Naslund et al. [1] reported that individuals use social media to seek mental health-related information and social support, which may facilitate access to assistance and provide resources that help them cope with psychological difficulties. Social media platforms also serve as spaces for building social connections and community belonging, both of which are important determinants of health [2,3]. On the other hand, exposure to social media may increase risks of privacy violations, cyberbullying, sleep disturbance, psychological distress, and even suicidal tendencies among adolescents [4]. In addition, Suhag and Rauniyar [5] found that the short videos posted by influencers on Instagram and TikTok, often portraying unrealistic “ideal” body types, can increase stress and contribute to eating disorders among adolescents. In short, social media is a double-edged sword: when used appropriately, it can facilitate access to reliable health information and support the formation of social networks and therefore promote well-being; when used improperly, it may have significant detrimental effects on well-being.

According to the World Health Organization (WHO), health is defined as “a state of complete physical, mental and social well-being and not merely the absence of disease or infirmity” (see WHO Constitution at https://www.who.int/about/governance/constitution, accessed on 9 July 2026). Therefore, understanding how social media use relates to health requires a comprehensive perspective that considers all three dimensions of health rather than focusing solely on mental, physical or social health. Against this background, an increasing number of review studies have been conducted in recent years to clarify how social media use relates to health from multiple perspectives (e.g., Paul and Headley-Johnson [6]). However, most existing reviews address one dimension of health in isolation, and few have simultaneously integrated all three WHO-defined dimensions, physical, mental and social well-being—with a single synthesizing framework. Therefore, this review aims to fill this gap.

Building upon the WHO framework, this review first summarizes current evidence regarding the associations between social media use and the three dimensions of health—mental health, physical health, and social health. We then review the emerging literature on health misinformation, an increasingly important issue in the social media era, before identifying research gaps and proposing directions for future research. Finally, we conclude by discussing how social media can be used in ways that promote well-being and by offering several policy recommendations. The search strategy, eligibility criteria, and synthesis approach used to identify the evidence base are described in the following section.

## 2. Review Methodology

### 2.1. Review Type and Rationale

This work adopts a narrative review approach to examine the relationship between social media use and health. A narrative review design [7,8,9] was selected because of the broad and multidisciplinary nature of the topic, which encompasses the three dimensions of health defined by the WHO—physical, mental, and social well-being—as well as the role of social media in health misinformation. The existing body of literature is highly heterogeneous with respect to study designs, populations, social media platforms examined, and health-related outcome measures. Such variability limits the feasibility and appropriateness of conducting a quantitative meta-analysis. Consequently, a narrative review provides a more suitable framework for synthesizing evidence across diverse research domains. This approach allows for a comprehensive and integrative examination of the literature, facilitating the identification of recurring patterns, areas of disagreement, and important knowledge gaps. Furthermore, it enables the exploration of complex interactions between social media use and health outcomes while highlighting key priorities for future research and policy development. A systematic or scoping review was not selected because this paper aims to integrate evidence, theory, and policy-relevant issues across multiple health domains rather than exhaustively map studies within a narrowly defined question.

### 2.2. Literature Search Strategy

A structured literature search was conducted between January and June 2026, with the final search update completed in August 2026, across major scholarly databases, including PubMed/MEDLINE, Web of Science (WoS), PubMed Central (PMC), Scopus, the Journal of Medical Internet Research (JMIR) portfolio, PLOS journals, Frontiers journals, and Google Scholar. The JMIR, PLOS, and Frontiers journal portfolios were searched directly to complement the broader database searches because these outlets frequently publish studies on digital health, health communication, infodemiology, and social media-related health research. To ensure comprehensive coverage across the thematic domains of this review—mental, physical, and social health, as well as misinformation—we applied eight keyword clusters independently and in combination. These clusters were designed to capture studies addressing social media use, health outcomes, and related behavioral or psychosocial mechanisms.

*Social media use* AND *mental health/depression/anxiety/psychological distress/subjective well-being*

*Social media use* AND *physical health/sleep quality/physical activity/sedentary behavior/screen time*

*Social media use* AND *social capital/social support/social isolation/loneliness/interpersonal relationships/connectedness*

*Social media use* AND *body image/eating disorders/disordered eating/social comparison*

*Social media use* AND *health misinformation/infodemic/fake news/COVID-19/health literacy/media literacy*

*Social media use* AND *health promotion/public health campaigns/behavior change/health communication*

*Problematic social media use/social media addiction/platform-specific effects/algorithmic amplification*

*Social media* AND *generative artificial intelligence/AI/health communication/deepfake*

Initial database queries returned approximately 6210 records in total. After deduplication and title screening, approximately 250 records remained for abstract and full-text assessment. An additional 15 sources were identified through manual screening of the reference lists of retrieved systematic reviews, scoping reviews, and meta-analyses. Key theoretical and foundational works predating the primary search window were also incorporated when they provided essential conceptual grounding—for example, research on the algorithmic dynamics of social media platforms [10], the diffusion of true and false information online [11], and the dose–response patterns between digital technology use and adolescent well-being [12]. The literature search and initial screening were conducted jointly by both authors. Any disagreements regarding source inclusion were resolved through discussion until consensus was reached.

After applying the eligibility criteria described in Section 2.3, a final corpus of 56 core references was retained for thematic synthesis. Additional theoretical and methodological sources were cited where necessary to support conceptual framing.

### 2.3. Eligibility Criteria

Sources were considered eligible for inclusion if they satisfied the following criteria:

- *Study type:* Peer-reviewed empirical studies (cross-sectional, longitudinal, and experimental designs), systematic reviews, scoping reviews, meta-analyses, narrative reviews, and reports from recognized public health or policy authorities (e.g., US Surgeon General [13], Pew Research Center [14]);
- *Publication period:* Sources published between 2015 and 2026 were prioritized. A small number of earlier foundational works were included where their theoretical or empirical contributions remain central to ongoing scholarly debate (e.g., [10,15]);
- *Language:* English; and
- *Topical relevance:* Direct examination of the relationship between social media use and physical, mental, or social health outcomes, or the role of social media in health misinformation, health promotion, or health communication

Sources were excluded if they focused exclusively on non-social-media digital technologies (e.g., broadcast television, video gaming) without reference to social media platforms, or if they were not available in peer-reviewed or recognized official report format.

### 2.4. Study Selection and Thematic Organization

Retrieved sources were screened for relevance based on title and abstract, followed by full-text review where necessary. A total of 56 references were retained and organized into five thematic domains corresponding to the paper’s structural sections:

1. Mental health—associations between social media use and depression, anxiety, psychological distress, subjective well-being, and emotional expression (for example [1,4,12,16,17,18,19,20,21,22,23]);
2. Physical health—associations with sleep quality, physical activity, sedentary behavior, and general physical health indicators (for example [24,25,26,27,28,29,30,31,32]), as well as platform-specific and content-specific effects on body image, social comparison, and disordered eating, particularly among adolescents and young adults (for example [5,33]);
3. Social health—social capital formation, social support, interpersonal relationships, online connectedness, and community belonging (for example [2,3,15,22,34,35,36,37]);
4. Health misinformation and infodemics—spread of false health information, algorithmic amplification, COVID-19-related misinformation, and media literacy interventions (for example [10,11,38,39,40,41,42,43,44,45]); and
5. Health promotion and future research directions—the role of social media in promoting health behaviors, and identified gaps and priorities for future investigation (for example [6,13,14,23,29,30,31,32]).

### 2.5. Synthesis Approach

A narrative synthesis approach was employed throughout this review, whereby findings from the included studies were summarized, compared, and interpreted thematically, with attention to both convergent and divergent evidence. Because the literature varied substantially in study designs, platforms examined, and outcome measures, statistical aggregation was not attempted. In areas where evidence was mixed or contested—such as the overall direction of social media’s effects on mental health, or the conditions under which social media use promotes versus undermines well-being—these divergent findings are explicitly acknowledged and incorporated into the paper’s future research agenda. Although the review draws on studies conducted across multiple countries and cultural contexts, it is noted that a substantial proportion of the existing literature originates from Western and East Asian settings, which may limit generalizability of the conclusions. To distinguish evidence synthesis from interpretation and future-oriented discussion, Section 3, Section 4, Section 5 and Section 6 summarize the current evidence base under the theme “What We Have Known So Far,” whereas Section 7 separately identifies unresolved questions and future research priorities under the theme “What We Need to Investigate in the Future.”

### 2.6. Weighting of Source Types

In selecting and synthesizing the included literature, priority was given to higher-order evidence. Systematic reviews, scoping reviews, and meta-analyses were weighted most heavily, as they provide synthesized findings across multiple primary studies. Large-scale longitudinal and cross-sectional empirical studies were prioritized over single-site or smaller observational studies. Narrative reviews, book chapters, and commentaries were included when they offered essential theoretical framing or addressed topics not yet covered by higher-order evidence. Reports from recognized public health authorities—including the US Surgeon General [13], the Pew Research Center [14], and the National Academies of Sciences, Engineering, and Medicine [28]—were treated as high-credibility grey literature given their rigorous evidence-synthesis processes. This weighting approach aligns with recommended practice for narrative reviews that integrate heterogeneous studies [7,8,9].

In cases where primary empirical studies overlapped with findings already summarized in systematic reviews, scoping reviews, or meta-analyses, we did not treat the same findings as separate independent evidence. Primary studies were cited when they provided specific empirical details, contextual examples, or platform-specific findings directly relevant to the narrative synthesis. Thus, the retained sources were used according to their evidentiary role rather than counted as equivalent units of evidence.

### 2.7. Limitations of the Search Process

Consistent with the narrative review design, the search and selection process described above does not conform to the full reporting standards of systematic or scoping reviews as outlined in PRISMA guidelines. Formal inter-rater reliability for study selection was not established, and the absence of pre-registration means that the review protocol was not independently verified prior to execution. These limitations are inherent to narrative reviews and are noted here in the interest of transparency. Readers seeking an exhaustive and fully reproducible synthesis of any single dimension of the topic—for example, the effects of social media on depression—are encouraged to consult the systematic and scoping reviews cited throughout this paper. Nevertheless, the narrative approach is appropriate for the present review because it allows integration of heterogeneous evidence across multiple health domains, theoretical traditions, and methodological approaches.

## 3. What We Have Known So Far About Social Media Use and Mental Health

Previous research has shown that social media use is associated with various mental health outcomes and that these associations may differ across types of social media platforms and according to users’ individual characteristics. Broadly speaking, social media users can be classified into active users, who actively engage with social media by posting content for specific purposes and interacting with others, and passive users, who primarily consume content by reading and viewing posts without specific purposes. In addition, growing evidence suggests that patterns of social media use and their implications for mental health differ depending on users’ motivations and personal backgrounds.

In particular, a substantial body of research and numerous review studies have focused on the relationship between social media use and the mental health of adolescents and young people, as concerns have been raised regarding the safety of social media use among these populations [13]. Such concerns are also shared by adolescents themselves [14]. Previous studies have reported that social media use is associated with depression, anxiety and psychological distress among young people [16]. However, findings from review studies suggest that these relationships are more complex than they initially appear. For example, Agyapong-Opoku et al. [17] reported that the impact of social media on adolescents’ mental health depends on individual characteristics and contextual factors and may result in both beneficial and detrimental outcomes. Woodward et al. also reported that the association between time spent on social media and mental health might depend on the social media platform used [18].

Using responses from 15,836 adults in the United Kingdom (UK), Yu et al. [19] found that heavy social media users exhibited more mental health problems, as measured using the General Health Questionnaire (GHQ-12), which assesses psychological distress and symptoms of anxiety and depression. Regression analyses indicated that individuals who frequently posted content were more likely to report more mental health problems after one year of social media use than those who did not post content. In addition, individuals who engaged in both active and passive social media use at high levels were found to experience more mental health problems than those who used social media infrequently.

Similarly, Jones et al. [20] reported a negative association between social media use and mental health among 425 university students in Australia. Their findings suggested that these associations varied across platforms. Overall, they observed a weak positive association between social media use and anxiety, whereas no significant associations were found with depression or stress. Moreover, Facebook use was weakly associated with more psychological distress, whereas TikTok use was weakly associated with poorer attentional control. Furthermore, Ye and Ho [21] used a two-wave panel survey conducted between May 2021 and May 2022 to examine the directional relationships between social media use and subjective well-being (SWB) among university students in Japan. They found that time spent on LINE, the most popular and frequently used social media platform for communication, predicted a decrease in participants’ levels of SWB one year later. On the other hand, they also found evidence of reverse directional relationships: participants’ (especially males’) levels of SWB negatively predicted their posting frequency on X (previously known as Twitter) one year later. Indeed, Ye and Ho further proposed an alternative approach to analyzing the associations between social media use and mental health by adopting a social media portfolio perspective [22]. Using data collected from 409 university students in Japan from August to September in 2022, they demonstrated that social media use portfolios—namely, X-only, LINE and X, Instagram and X, and Discord and X—were associated with increased loneliness through perceptions of physical health as a mediator. In a subsequent study based on responses from 462 university students collected in June, 2024, Ye and Ho [23] examined two social media use portfolios: users who concurrently used LINE, X and Instagram, and users who concurrently used LINE, X, Instagram and Discord. Their findings revealed that, among users in the former group, their time spent on LINE and Instagram was associated with increased depressive tendencies (DT) through a mediating factor of headaches, while their time spent on X and posting frequency were directly associated with increased DT at a marginally significant level. In contrast, among users in the latter group, time spent on X and posting frequency were indirectly associated with increased DT through headache symptoms as a mediator, and their posting frequency on Instagram was associated with increased DT at a marginally significant level. Taken together, the findings of Ye and Ho [21,22,23] further suggested that the relationship between social media use and mental health should be examined not only at the level of individual platforms but also from a social media platform portfolio perspective, and from different perspectives of mental health (e.g., SWB as a positive side, and loneliness and DT as negative sides). Considering the combinations of platforms used by individuals with different personal factors may provide a more comprehensive understanding of how social media use is associated with mental and physical health outcomes. In addition, a narrative review conducted by Hall [46] reported that social media use may improve belongingness but not relate to loneliness.

The social media portfolio perspective can be situated within several established theoretical traditions in communication and health behavior research. First, media repertoire theory argues that individuals routinely engage with multiple media channels, and that the configuration of these channels—rather than any single medium—shapes patterns of information exposure, social interaction, and psychological outcomes. A social media portfolio represents the platform-specific extension of this repertoire logic, reflecting how users assemble and navigate combinations of social media environments [47,48]. Second, uses and gratifications theory suggests that people select media based on diverse motivations—such as information seeking, social connection, entertainment, or self-expression—and that different media channels afford distinct gratifications [49,50]. From this standpoint, a user’s social media portfolio captures the motivational structure underlying platform choices, as different platforms serve different psychological and social needs. Third, research in health communication and health behavior emphasizes that health-related attitudes and behaviors emerge from exposure to multiple, interacting information sources rather than isolated channels [51,52]. A portfolio approach therefore aligns with theoretical expectations that cross-platform dynamics shape health outcomes. Taken together, these traditions provide a conceptual foundation for the portfolio perspective and clarify why it represents a meaningful advance over single-platform analyses, which may oversimplify the complex, multi-platform nature of contemporary social media use.

Table 1 below summarizes some of the key findings of recent studies.

## 4. What We Have Known So Far About Social Media Use and Physical Health

Although the relationship between social media use and mental health has received considerable attention, growing evidence suggests that social media use is also closely associated with physical health. Unlike mental health outcomes, which are often directly associated with online interactions and emotional experiences, physical health outcomes are more likely to be affected and observed through changes in health-related behaviors, such as physical activity, sleep, dietary habits, and sedentary lifestyles. Consequently, previous studies have increasingly investigated the mechanisms through which social media use relates to physical health, while also recognizing that social media may serve as both a health-promoting and a health-compromising tool.

One of the most frequently studied physical health outcomes associated with social media use is sleep. Previous research has consistently shown that excessive social media use is associated with poor sleep quality, shorter sleep duration, delayed bedtimes, and daytime fatigue, particularly among adolescents and young adults [24,25]. Several mechanisms have been proposed to explain these relationships. First, prolonged social media use may displace sleep time, resulting in insufficient sleep. Second, exposure to emotionally arousing or highly engaging online content before bedtime may increase psychological arousal and make it more difficult to fall asleep. Third, blue-light exposure from smartphones and other digital devices may interfere with circadian rhythms and suppress melatonin secretion, thereby reducing sleep quality [25]. Because sleep plays a critical role in maintaining both physical and mental health, these findings suggest that sleep disturbance may represent an important pathway linking social media use to broader health outcomes.

Another important concern is the relationship between social media use and physical activity. Previous studies have generally reported that more social media use is associated with lower levels of physical activity and increased sedentary behavior [24,26]. Time spent using social media may replace time that could otherwise be devoted to exercise or other physically active pursuits. As a result, prolonged social media use may contribute to a more sedentary lifestyle, which has been linked to obesity, cardiovascular disease, metabolic disorders, and other chronic health conditions. Nevertheless, recent reviews have emphasized that this relationship is not entirely negative. Social media platforms can also encourage physical activity by providing exercise-related information, online fitness communities, and peer support that motivate individuals to adopt healthier lifestyles [6,26]. Therefore, the associations between social media and physical activity appear to depend on how social media is used rather than simply how long it is used.

In addition to physical activity and sleep, recent studies have examined how social media use is related to other physical health indicators. For example, Ye and Ho [22,23] investigated the associations between social media use and both mental and physical health among university students in Japan using a social media portfolio perspective. Their findings showed that regardless of different social media use patterns, their usage was associated with higher levels of loneliness, which was mediated by poorer perceptions of physical health [22]. In addition, headache symptoms played a mediating role in the relationship between the use of different combinations of social media platforms and depressive tendency. Specifically, among students who used LINE, X, and Instagram, time spent on LINE and Instagram was significantly indirectly associated with increased depressive tendency through headache symptoms, whereas time spent on X and posting frequency showed direct associations with depressive tendency at a marginally significant level. In contrast, among students who additionally used Discord, time spent on X and posting frequency were significantly indirectly associated with increased depressive tendency through headache symptoms [23]. These findings suggest that physical symptoms should not be regarded merely as outcomes of social media use but also as important mechanisms through which social media use may influence mental health.

More broadly, previous reviews have suggested that the relationship between social media use and physical health is highly complex. On one hand, problematic or excessive social media use has been associated with unhealthy lifestyles, reduced physical activity, poor sleep, musculoskeletal discomfort, visual fatigue, headaches, and other adverse physical symptoms [24,27,28]. Furthermore, prior research has also reported the association between high social media use and more body image concerns and eating disorders [53,54]. On the other hand, social media has become an increasingly valuable tool for health promotion. Health organizations, healthcare professionals, and public health agencies have used social media to disseminate health information, promote healthy behaviors, increase public engagement, and provide social support for individuals managing chronic diseases [29,30,31,32]. Online health communities also enable users to exchange experiences, receive encouragement from peers, and obtain practical advice for maintaining healthier lifestyles. Therefore, social media should not simply be regarded as a risk factor for poor physical health but also as a potentially effective platform for promoting healthy behaviors when used appropriately.

Taken together, the current literature suggests that social media use has both direct and indirect associations with physical health. Rather than exerting uniformly beneficial or harmful effects, its influence appears to depend on multiple factors, including the specific social media platforms used, users’ behavioral patterns, the content they are exposed to, and their individual characteristics. Furthermore, emerging evidence indicates that physical health and mental health are closely interconnected in the context of social media use. Physical health indicators, such as sleep quality and headache symptoms, may function as mediators linking social media use to psychological well-being, suggesting that future research should consider mental and physical health simultaneously rather than as separate outcomes. Table 2 summarizes the key findings of the relationship between social media use and physical health issues.

## 5. What We Have Known So Far About Social Media Use and Social Health

According to the WHO, health encompasses not only physical and mental well-being but also social well-being. Social health generally refers to individuals’ ability to establish and maintain meaningful interpersonal relationships, participate in society, and obtain adequate social support. As social media was originally designed to facilitate communication and social interaction, understanding its relationship with social health is essential for developing a comprehensive understanding of how social media influences overall health.

One of the most frequently studied components of social health is social support. Previous studies have suggested that social media provides users with opportunities to maintain existing interpersonal relationships, establish new social connections, and obtain emotional and informational support from family members, friends, and online communities. These online interactions may help individuals reduce feelings of isolation, cope with stressful situations, and strengthen their sense of belonging, particularly among those who have limited opportunities for face-to-face communication.

Gilmour et al. [2] systematically reviewed empirical studies examining how Facebook facilitates social support and how this support relates to health outcomes. Across 33 studies, the authors find that Facebook can serve as a meaningful source of emotional, informational, and instrumental support, particularly for individuals managing health challenges, stigmatized conditions, or limited offline support networks. Participation in Facebook groups and health-related communities often enhances feelings of belonging, reduces isolation, and increases perceived social support. These benefits are most evident when interactions are active, reciprocal, and embedded within supportive communities. However, the review also highlights important limitations. Not all Facebook interactions are supportive, and some studies report mixed or null associations between Facebook use and health. Methodological weaknesses—such as cross-sectional designs, inconsistent definitions of “social support,” and reliance on self-reported Facebook activity—limit causal conclusions. Overall, the authors concluded that Facebook has the potential to promote social support and improve well-being, but effects depend on user motivations, community norms, and the quality of interactions rather than mere platform use.

Recent empirical studies have further demonstrated that social media can facilitate the acquisition of social support, although the extent of these benefits depends on users’ communication patterns, individual characteristics, and the duration of use. For example, Ye and Ho [34] examined university students in Japan before and during the COVID-19 pandemic and investigated the relationships among generalized trust, Twitter use, social support, and SWB. Their findings indicated that before the COVID-19 pandemic (i.e., 2019), Twitter usage was associated with the formation of social networks with relatively close others, which led to increased social support from these people; however, such social support was not associated with their SWB. On the other hand, during the COVID-19 pandemic (i.e., 2020), the total social support received from Twitter was associated with an increased level of well-being, whereas satisfaction with social networks via Twitter was associated with a decreased level of well-being. These findings suggest that the relationship between social media use and social well-being depends on how individuals develop and maintain supportive interpersonal relationships through social media.

Another important concept related to social health is social capital, which refers to the resources that individuals obtain through their social relationships and social networks. Previous studies have suggested that social media has the potential to facilitate the development of social capital by expanding communication networks, maintaining interpersonal relationships across geographical boundaries, and increasing opportunities for social participation. However, evidence also indicates that different forms of social capital may have different associations with health outcomes.

Antheunis et al. [15] systematically reviewed empirical studies on Facebook use and social capital and concluded that Facebook use was generally associated with greater bridging social capital, which refers to access to new information and broader social networks. By contrast, evidence regarding bonding social capital, which reflects emotional support derived from close interpersonal relationships, was less consistent. Their review further suggested that the relationship between Facebook use and social capital depends on users’ motivations, patterns of use, and the quality of their online interactions rather than simply the amount of time spent on the platform. In addition, Liu et al. [35] reported that excessive or passive social media use—particularly when it occurs without meaningful interaction—may be associated with social isolation. These findings suggest that social media should not be viewed as uniformly beneficial or harmful for social health, as different forms of social capital may emerge from different patterns of social media use.

Similarly, Ryan et al. [3] reviewed the literature on social media and social connectedness and argued that online social interactions exert both positive and negative influences on social well-being. On the positive side, social media enables users to maintain interpersonal relationships, communicate with geographically distant friends and family members, and strengthen their sense of belonging to online communities. These opportunities may facilitate the development of both bonding and bridging social capital. On the other hand, excessive reliance on online interactions has been associated with reduced face-to-face communication, increased feelings of loneliness, and more superficial interpersonal relationships. The authors therefore concluded that current evidence does not support the view that social media is either inherently beneficial or harmful to social health; instead, its effects depend largely on how individuals use social media and integrate online interactions with offline social relationships.

Recent studies have further demonstrated that different social media platforms may facilitate different forms of social capital. Using survey data from users of Facebook, Twitter, Instagram, and Snapchat, Phua et al. [36] examined how platform preferences were associated with bonding and bridging social capital. Their findings showed that Snapchat users reported the highest levels of bonding social capital, reflecting stronger emotional ties with close friends, whereas Twitter users exhibited higher levels of bridging social capital through broader and more diverse social networks. Facebook and Instagram demonstrated characteristics of both forms of social capital, although their associations varied according to users’ communication purposes. These findings suggest that different social media platforms serve distinct social functions and reinforce the importance of adopting a platform-specific perspective when examining the relationship between social media use and social health.

Consistent with these findings, Ye and Ho [22] examined the relationships among personality traits, face-to-face social capital, online social capital, and health among university students in Japan. Their findings indicated that face-to-face social capital was positively associated with better mental health (higher SWB and lower loneliness) and greater perceived physical health, and also facilitated the development of online social capital. In contrast, although social media use was associated with increased online social capital, online social capital itself was not associated with either mental health or perceived physical health. These findings suggest that online social relationships may complement—but not replace—face-to-face social interactions, highlighting the continuing importance of offline social capital for promoting overall well-being.

Overall, existing evidence suggests that social media use can promote social health by facilitating social support, strengthening interpersonal relationships, and fostering both bonding and bridging social capital. However, these associations vary considerably according to the characteristics of individual users, their communication patterns, and the social media platforms they use. Furthermore, although online social relationships may complement offline interpersonal relationships, current evidence suggests that face-to-face social relationships may remain particularly important sources of social capital for promoting physical, mental, and social well-being. More research is necessary to clarify how offline and online social capital interact across different populations and contexts [37].

Table 3 summarizes the key findings of the relationship between social media use and social health issues.

## 6. What We Have Known So Far About Social Media Use and Misinformation

### 6.1. Social Media as a Major Source of Health Information

Social media has fundamentally transformed the way people seek, obtain, and exchange health information. Unlike traditional media, social media platforms allow users not only to access health-related information but also to generate, modify, and disseminate content to large audiences in real time. Consequently, social media has become an important channel through which individuals obtain health knowledge, communicate with healthcare professionals, and exchange experiences with other users [29,30,31,32].

The increasing accessibility of social media has also created new opportunities for health promotion. Public health organizations, healthcare professionals, and researchers have increasingly adopted social media to disseminate evidence-based health information, promote healthy lifestyles, improve health literacy, and encourage public engagement in disease prevention [29,30,31,32]. Compared with conventional health communication, social media enables bidirectional communication, allowing users to ask questions, receive immediate feedback, and participate in online health communities. These characteristics have substantially expanded the reach of health education and public health campaigns. However, the same characteristics that facilitate rapid dissemination of health information also enable false or misleading information to spread widely. Because information on social media is often generated by users with diverse backgrounds and varying levels of expertise, the quality and credibility of health-related content are not always guaranteed. Consequently, distinguishing reliable information from misinformation has become an increasingly important public health challenge.

### 6.2. Health Misinformation and the COVID-19 Infodemic

The widespread use of social media has substantially increased the speed and scale at which health information is disseminated. However, it has also facilitated the rapid spread of health misinformation. Even before the COVID-19 pandemic, researchers [11] had already reminded us of the spread of medical-related rumors back in 2018 using data obtained from Twitter between 2006 and 2017. Indeed, during the COVID-19 pandemic, misleading information regarding the origins of the virus, modes of transmission, preventive measures, vaccines, and treatments spread extensively across multiple social media platforms, often reaching large audiences before evidence-based information became available [38,39,40,41,42]. This overwhelming amount of both accurate and inaccurate information prompted the WHO to introduce the term *infodemic*, referring to the overabundance of information that makes it difficult for individuals to identify trustworthy sources and reliable guidance [43].

Recent reviews have consistently highlighted health misinformation as one of the major public health challenges associated with social media use [38,39,42]. Exposure to false health information has been associated with increased uncertainty, reduced trust in healthcare professionals and public health authorities, vaccine hesitancy, and lower adherence to recommended preventive behaviors [38,41,42]. Consequently, health misinformation has become not only a communication problem but also an important determinant of individual and population health.

### 6.3. Why Does Health Misinformation Spread on Social Media?

Previous studies suggest that both technological and human factors drive the rapid spread of health misinformation. From a technological standpoint, the design of social media platforms allows users to create, share, and redistribute information almost instantaneously. Recommendation algorithms further amplify popular or emotionally engaging content—regardless of its accuracy—making it more likely for misinformation to reach large audiences [10]. In addition, users are frequently exposed to information that aligns with their existing interests and beliefs, creating so-called “echo chambers” that reinforce prior opinions while limiting exposure to alternative perspectives. Combined with the “hostile media effect” and the empowering nature of social media, which allows individuals to publish their own interpretations of available information, these dynamics contribute to the proliferation of health misinformation on social media [44]. From a human perspective, the formation and dissemination of health misinformation are influenced by users’ cognitive, psychological, and social characteristics. Ho and Ye [45] proposed that false health information is generated through interactions between information providers and information receivers. On the one hand, misinformation may originate from misunderstanding scientific evidence, commercial interests, ideological motivations, or intentional disinformation. On the other hand, users’ health literacy, social media literacy, prior beliefs, emotions, and critical thinking skills influence how they interpret, evaluate, and further disseminate health information.

Evidence also suggests that misinformation spreads differently from factual information. Using approximately 126,000 news stories shared on Twitter over 11 years, Vosoughi et al. [11] demonstrated that false news spreads significantly faster, farther, and more broadly than truthful news. They argued that false information is often perceived as more novel and emotionally engaging, making users more likely to share it with others. These findings suggest that the rapid spread of misinformation is attributable not only to platform algorithms but also to human behavioral tendencies.

### 6.4. Strategies for Reducing Health Misinformation

Given the substantial influence of health misinformation on public health, increasing attention has been devoted to developing effective intervention strategies. Previous reviews have suggested that no single intervention is sufficient; rather, reducing health misinformation requires coordinated efforts from governments, healthcare professionals, researchers, educators, and social media platforms [38,41,42], via strategies focusing on communication, technology, and multimedia [55].

One of the most commonly discussed interventions is fact-checking or debunking, which involves the post hoc correction of false claims, typically conducted by independent organizations, platform-embedded labels, or crowdsourced flagging systems. Although theoretically fact-checking/debunking is a useful method, it has its limitations. Ho et al. [44] noted that individuals may selectively consult only those sources they already trust when attempting to verify information, which can lead to biased outcomes. In politically polarized or low-trust information environments, fact-checking may be less effective because individuals often evaluate corrective information through the lens of prior beliefs, institutional trust, and perceived source credibility [56]. Such cases create challenges for members of the public who lack sufficient health literacy to evaluate the accuracy of competing directives—for example, choosing between guidance from political authorities and recommendations from medical experts.

Another intervention is *inoculation* or *prebunking*. This approach represents a theoretically grounded alternative, drawing on psychological inoculation theory to expose individuals to weakened forms of misinformation together with explicit refutation strategies before exposure occurs. It functions as a proactive defensive measure designed to build resistance to misinformation [45].

One promising approach is to improve users’ health literacy and social media literacy. Health literacy enables individuals to understand and evaluate health information, whereas social media literacy helps users critically assess online content, identify unreliable information sources, and make informed decisions regarding information sharing. Ho and Ye [45] further argued that strengthening social media literacy can reduce individuals’ susceptibility to false health information and decrease its subsequent dissemination.

In addition to educational interventions, platform-level strategies have also attracted considerable attention. Social media companies have introduced fact-checking systems, warning labels, content moderation, and algorithmic adjustments to reduce the visibility of false health information. Although these measures have shown some effectiveness, systematic reviews indicate that their impacts remain inconsistent and that misinformation continues to spread rapidly, particularly during emerging public health crises [41,42].

Taken together, the existing literature indicates that combating health misinformation requires both individual- and platform-level interventions. Improving users’ abilities to critically evaluate online information, together with developing more transparent recommendation algorithms and evidence-based content moderation strategies, may substantially reduce the spread and influence of health misinformation. Nevertheless, as social media technologies continue to evolve, identifying effective and sustainable approaches to mitigating misinformation remains an important challenge for researchers and policymakers.

It is also important to distinguish between interventions that have been empirically evaluated and those that remain mainly conceptual or policy-oriented. Fact-checking, warning labels, prebunking/inoculation, and media literacy interventions have been examined to varying degrees, although their effects are context-dependent. By contrast, algorithmic transparency, platform governance, and AI-based misinformation detection remain promising but require stronger real-world evaluation.

### 6.5. The Emerging Threat of Generative AI in Health Misinformation

The recent rapid development of generative artificial intelligence (generative AI) has created new opportunities for producing and disseminating health misinformation. Unlike traditional misinformation—which is typically created and spread by human actors—generative AI is capable of producing highly convincing false health claims, fabricated medical evidence, and synthetic media at unprecedented scale and speed, as large language models and image-generation tools of generative AI have substantially lowered the technical barriers to creating believable false health information [57].

The implications for health communication are significant. Murphy-Reuter and Burke-Garcia [58] argue that the same generative AI technologies that pose misinformation risks also offer new opportunities for health communicators—using generative AI to support them in creating well-sourced and unbiased health information for the community.

From a public health perspective, the generative AI misinformation challenge intersects with all of the intervention categories mentioned above. Existing fact-checking infrastructure is ill-equipped to handle the volume of AI-generated content; inoculation strategies must be updated to address synthetic media specifically; and platform moderation systems require new AI-detection capabilities that are themselves subject to adversarial circumvention. The governance of AI-generated health misinformation therefore represents one of the most urgent and underexamined priorities at the intersection of social media, health, and digital policy [57,58].

Table 4 summarizes the key findings of the relationship between social media use and health misinformation.

## 7. What We Need to Investigate in the Future

Although a growing body of literature has enhanced our understanding of the relationships between social media use and human health, several important gaps remain. Existing findings consistently suggest that social media functions as a double-edged sword, producing both beneficial and detrimental effects on mental health, physical health, and health information environments. Nevertheless, many unanswered questions remain regarding causal mechanisms, individual differences, platform characteristics, and effective intervention strategies. Future research should therefore adopt more rigorous and interdisciplinary approaches to address these challenges.

### 7.1. Moving Beyond Cross-Sectional Research Toward Causal Explanations

One major limitation of the current literature is the heavy reliance on cross-sectional designs. Although many studies have reported significant associations between social media use and health outcomes, such findings do not necessarily establish causality. For example, social media use may lead to poorer mental health, but individuals experiencing loneliness, depression, or psychological distress may also increase their social media use as a coping strategy. Similar bidirectional relationships have been observed in recent longitudinal studies examining different social media use and subjective well-being among university students in Japan [21].

Future research should therefore place greater emphasis on longitudinal panel studies, cross-lagged analyses, natural experiments, and randomized controlled trials whenever feasible. Such approaches would allow researchers to disentangle the directionality of effects and identify the causal pathways linking social media use to health outcomes. Understanding whether social media serves primarily as a cause, consequence, or amplifier of health problems remains a critical challenge for future research.

A holistic analytical framework can help clarify how different types of evidence contribute to understanding the relationship between social media use and health. Associational analyses identify correlations between variables but cannot determine temporal ordering. Temporal prediction models, including cross-lagged analyses, assess whether earlier social media use predicts later health outcomes (or vice versa), thereby establishing directionality without confirming causality. Statistical mediation analyses examine the mechanisms through which social media may influence health, identifying intermediate variables such as sleep quality, headache symptoms, or content exposure. Finally, causal inference approaches—including natural experiments and randomized controlled trials—provide the strongest evidence by isolating the effects of specific exposures or interventions. Together, these analytical strategies form a complementary toolkit: associations reveal patterns, temporal models establish ordering, mediation clarifies mechanisms, and causal designs identify true effects. Future research should integrate these approaches to build a cumulative and mechanistically informed understanding of how social media influences mental, physical, and social health.

### 7.2. Understanding Individual Differences and Vulnerable Populations

The heterogeneous findings reported across previous studies suggest that social media does not affect all users equally. The Differential Susceptibility to Media Effects Model (DSMM) proposes that media effects depend on users’ dispositional, developmental, and social characteristics [12]. Consistent with this perspective, previous studies have shown that age, gender, personality traits, health literacy, and existing mental health conditions may influence how individuals respond to social media use.

Future research should therefore investigate which groups are particularly vulnerable to adverse health consequences. More attention should be paid to gender differences, given accumulating evidence that females and males may experience different psychological and behavioral impacts from social media exposure. Similarly, recent research on eating disorders suggests that social media may affect body image concerns differently across gender and sexual orientation groups [59]. Future studies should also examine whether adolescents, older adults, and individuals with chronic health conditions differ in their susceptibility to problematic social media use, misinformation exposure, and health-related behavioral changes. In addition, prior research has sparsely studied the relationship between social media use and the health of older adults, non-student young adults, low-income populations, and users in the Global South. Therefore, future research should examine this relationship with more demographically and culturally diverse samples.

### 7.3. Examining Social Media Content Rather than Usage Time Alone

Many previous studies have measured social media use primarily through time spent on platforms. However, studies have shown that time spent on platforms and the posting frequency on them have different associations with mental and physical health, even considering cultural differences [23,60]. Additionally, emerging evidence suggests that content exposure and usage motivations may be more important than duration alone. For instance, exposure to appearance-focused content may contribute to body dissatisfaction and eating disorders [33,61], whereas participation in health-support communities may improve health literacy and social support [29,30].

Future studies should move beyond simple screen-time measures and examine what types of content users consume, create, and share. Researchers should investigate how different forms of content—including health information, fitness content, political discussions, influencer-generated content, and misinformation—affect users’ mental and physical health. Such approaches may help explain why some users experience positive outcomes while others experience adverse consequences despite similar levels of usage.

### 7.4. Incorporating Objective Behavioral Measures

Another limitation of the current literature is the widespread reliance on self-reported measures of social media use. Self-reports are vulnerable to recall bias and may not accurately reflect actual behavior. Several studies have demonstrated discrepancies between self-reported and objectively recorded social media use [20].

Future research should integrate objective behavioral measures, including digital trace data, smartphone usage logs, ecological momentary assessment, wearable-device data, and platform-generated analytics. Combining these objective measures with traditional survey data would provide a more accurate understanding of how specific behaviors influence health outcomes. Such approaches may also clarify whether problematic outcomes are attributable to usage intensity, specific behavioral patterns, or particular platform features.

### 7.5. Adopting a Social Media Portfolio Perspective

Most previous studies have focused on individual social media platforms in isolation. However, contemporary users rarely rely on a single platform. Instead, they typically use multiple social media platforms simultaneously to fulfill different communication and information needs. Recent studies adopting a social media portfolio perspective have demonstrated that combinations of platforms are associated with different mental and physical health outcomes [22,23,62].

Future research should therefore investigate how multiple-platform use shapes health outcomes. Researchers should examine how different platform portfolios interact with user characteristics, social capital, health behaviors, and psychological well-being. Such an approach may provide a more realistic understanding of contemporary social media ecosystems and improve the development of targeted health interventions.

### 7.6. Understanding the Interconnections Between Mental and Physical Health

Current research often treats mental health and physical health as separate domains. However, growing evidence suggests that they are closely intertwined in the context of social media use. For example, sleep disturbances and headache symptoms may serve as mediating mechanisms linking social media use to loneliness, depressive tendencies, and reduced subjective well-being [22,23].

Future studies should adopt integrated models that simultaneously examine physical and mental health outcomes. Particular attention should be paid to mediating and moderating mechanisms involving sleep quality, physical activity, sedentary behavior, musculoskeletal symptoms, and perceived health status. Understanding these complex pathways may provide more comprehensive insights into how social media influences overall well-being.

### 7.7. Developing Effective Interventions Against Health Misinformation

Although health misinformation has been widely recognized as a major public health threat, evidence regarding effective interventions remains limited. Existing approaches—including fact-checking, warning labels, content moderation, and algorithmic adjustments—have produced mixed results [41,42].

Future research should evaluate the long-term effectiveness of these interventions and identify the conditions under which they work best. In addition, more research is needed to examine how health literacy, social media literacy, critical thinking skills, and trust in scientific institutions influence individuals’ susceptibility to misinformation. Given the rapid development of AI technologies, several recent studies are reporting that generative AI may, on the one hand, contribute to the creation of health misinformation [57], and, on the other hand, serve as a possible tool for fighting the infodemic [58]. Therefore, future studies should also examine how AI-generated content may influence the production and dissemination of health misinformation on social media.

### 7.8. Addressing Social Polarization and Societal Impacts

Beyond individual health outcomes, social media increasingly influences broader societal processes, including polarization, trust, and public discourse. Recommendation algorithms and online echo chambers may reinforce existing beliefs and increase divisions among social groups [10,63]. Such processes may further contribute to resistance to public health recommendations and reduced trust in scientific evidence.

Future research should therefore investigate how social media ecosystems shape societal health outcomes at both individual and collective levels. Understanding the relationships among algorithmic exposure, misinformation, polarization, and health-related decision-making will become increasingly important in future digital societies.

### 7.9. Toward an Interdisciplinary Research Agenda

Finally, the complexity of social media and health relationships requires collaboration across multiple disciplines, including public health, psychology, communication studies, information science, medicine, computer science, and policy studies. So far, scholars have called for a public health research agenda on social media, particularly with respect to mental and physical health [64], as well as more interdisciplinary approaches to understanding social media’s impact on young people’s well-being [65]. Building on these calls, the priorities outlined above suggest that future research should integrate theoretical perspectives and methodological approaches from public health, psychology, communication studies, information science, computer science, and policy studies.

Ultimately, the goal of future research should not be merely to identify the risks of social media use but also to understand how social media can be designed, regulated, and used in ways that maximize its health-promoting potential while minimizing its associated harms.

Taken together, these nine research priorities point toward several overarching directions that warrant urgent attention. First, future work must move beyond cross-sectional associations and adopt longitudinal, experimental, and causal designs capable of clarifying the mechanisms through which social media influences mental, physical, and social health. Second, researchers should prioritize integrative approaches that examine individual differences, platform portfolios, content exposure, and the interconnected pathways linking mental and physical health, rather than treating these domains in isolation. Third, the rapid evolution of social media environments—including algorithmic personalization, platform interoperability, and the increasing presence of generative AI—requires timely empirical investigation, as these shifts may fundamentally reshape exposure patterns, misinformation dynamics, and societal polarization. The remaining priorities reinforce these core directions by emphasizing the need for diverse samples, objective behavioral measures, content-specific analyses, and interdisciplinary collaboration across public health, psychology, communication studies, information science, and computer science. Collectively, these synthesized priorities provide a structured roadmap for advancing a cumulative, interdisciplinary, and policy-relevant evidence base on social media and health.

Table 5 summarizes the proposed future research agenda on the relationship between social media use and health.

### 7.10. Limitations of This Review

Several limitations should be noted. First, because this article is a narrative review rather than a systematic or scoping review, the search process was structured but not exhaustive, and the review was not preregistered. Second, some conclusions are based on limited or uneven evidence across populations, platforms, and health outcomes, and should therefore be interpreted as preliminary. Third, the included studies vary in design, measurement, population, and cultural context, limiting direct comparability. Finally, because social media platforms, algorithms, and generative AI are rapidly evolving, some evidence may become outdated quickly.

## 8. Conclusions

Social media has become an indispensable component of contemporary society and exerts profound influences on how people communicate, access information, and manage their health. This review synthesized recent evidence regarding the relationships between social media use and human health from the following perspectives: mental health, physical health, social health and health misinformation. Overall, the findings consistently indicate that social media is neither inherently beneficial nor inherently harmful. Rather, its impact depends on how it is used, by whom it is used, and the social and technological environments in which it operates.

With regard to mental health, previous studies have demonstrated associations between social media use and a variety of psychological outcomes, including subjective well-being, loneliness, anxiety, depression, psychological distress, and social isolation. At the same time, social media can provide valuable opportunities for social support, community building, and access to mental health resources. Emerging evidence further suggests that these relationships vary according to users’ characteristics, motivations, patterns of use, and combinations of platforms. Consequently, understanding social media use requires moving beyond simplistic assumptions that more use is necessarily harmful and recognizing the complexity of users’ experiences across different social media ecosystems.

Regarding physical health, the current literature suggests that social media use influences health-related behaviors and symptoms through multiple pathways. Excessive or problematic use has been linked to sleep disturbance, reduced physical activity, sedentary behavior, headaches, visual fatigue, and other health concerns. However, social media also provides important opportunities for health promotion by facilitating access to health information, peer support, online health communities, and behavior-change interventions. Furthermore, evidence increasingly indicates that physical health and mental health are closely interconnected, with physical symptoms such as sleep problems and headaches potentially serving as mechanisms linking social media use to psychological/subjective well-being.

Regarding social health, existing evidence suggests that social media has considerable potential to strengthen interpersonal relationships by facilitating social support, maintaining communication with geographically dispersed family members and friends, and fostering both bonding and bridging social capital. Nevertheless, these benefits are not universal. Their magnitude depends on users’ communication behaviors, the quality of online interactions, and the specific social media platforms used. Importantly, current evidence consistently indicates that online social relationships complement rather than replace face-to-face interpersonal relationships. Offline social capital may remain an especially important foundation for promoting physical, mental, and social well-being, highlighting the need to maintain a healthy balance between online and offline social interactions.

The review also highlighted the growing public health challenge posed by health misinformation. The COVID-19 pandemic demonstrated how social media can accelerate the spread of both accurate and inaccurate health information, contributing to large-scale infodemics. Recommendation algorithms, echo chambers, emotional engagement, and individual cognitive factors collectively facilitate the dissemination of misinformation. Although educational initiatives, fact-checking systems, and platform-level interventions have shown promise, their effectiveness remains inconsistent, indicating the need for continued research and policy innovation.

Taken together, the existing evidence suggests that the health consequences of social media use arise from complex interactions among technological, psychological, behavioral, and social factors that jointly shape physical, mental, and social well-being. Future research should therefore move beyond cross-sectional approaches and adopt longitudinal, experimental, and interdisciplinary frameworks. Greater attention should also be devoted to objective behavioral measures, individual differences, platform-specific social media portfolio perspectives, and the development of effective interventions that promote healthy use while reducing harm. Future studies should further extend this evidence base to more diverse age groups, socioeconomic backgrounds, and cultural contexts. At the same time, researchers should continue to investigate how social media contributes not only to individual health outcomes but also to broader societal phenomena, including misinformation, trust, and social polarization.

Overall, the existing literature suggests that social media is neither inherently beneficial nor harmful to health. Rather, its influence depends on how, why, and under what circumstances it is used. Following the WHO definition of health, future research should move beyond examining isolated health outcomes and adopt a more integrated perspective that simultaneously considers physical, mental, and social well-being. Such an approach will provide a more comprehensive understanding of how social media use influences human health across multiple dimensions. Ultimately, maximizing the health-promoting potential of social media while minimizing its associated risks will require collaborative efforts among researchers, healthcare professionals, educators, policymakers, technology companies, and social media users themselves to create healthier and more trustworthy digital societies.

## Figures and Tables

**Table 1:** Key Findings of the Relationship between Social Media Use and Mental Health Issues.

Data Source	Country	Findings	References
Collected between 2020 and 2022 from 15,836 adults	UK	✧ Frequent users had more mental health issues compared with non-frequent users (i.e., never posted). ✧ Frequent users who also viewed more messages had more mental health issues compared with those users who rarely viewed or posted on social media.	[19]
Collected from 425 university students	Australia	✧ Social media use had a small association with anxiety. ✧ Facebook use had a weak association with psychological distress, and TikTok use had a weak association with attentional control, which the authors suggested might be overstated.	[20]
Collected from 647 university students who participated in both surveys	Japan	✧ Time spent on LINE predicted participants’ decreased SWB a year later. ✧ Participants’ (especially males’) SWB predicted decreased posting frequency on X (Twitter) a year later.	[21]
Collected from 409 university students	Japan	✧ Social media use was indirectly associated with loneliness, which was mediated by perceived physical health among four different social media portfolios studied, i.e., X only, LINE and X, Instagram and X, and Discord and X.	[22]
Collected from 462 university students	Japan	✧ In the group of students who used LINE, X and Instagram, LINE and Instagram usage time were indirectly associated with DT, with headache symptoms as a mediator, while time spent on X and posting frequency were directly associated with DT at a marginally significant level. ✧ In the group of students who used LINE, X, Instagram and Discord, their time spent on X and posting frequency were indirectly associated with DT through headache symptoms as a mediator, and posting frequency on Instagram was directly associated with DT at a marginally significant level.	[23]

**Table 2:** Key Findings of the Relationship between Social Media Use and Physical Health Issues.

Data Source	Country	Findings	References
***Sleep Disturbance***			
Collected from 43,994 Middle School and High School students (8th, 10th, and 12th grades)	USA	✧ The association between social media use and sleep differed by baseline sleep status: Increased social media use was associated with poorer sleep quality among adolescents with adequate sleep, but with better sleep quality among those with sleep deprivation.	[24]
A summary of research findings between 2020 and 2023 from MEDLINE	Various	✧ From results obtained from 17 cross-sectional studies, 16 of them reported that different dimensions of social media use were associated with poor sleep. The remaining one showed that social media use did not have a significant association with sleep on average, but did have a negative association with those young people with depressive symptoms. ✧ Two cohort studies reported that high levels of social media use were associated with shorter sleep duration in the future.	[25]
***Physical Activities***			
Collected from 43,994 Middle School and High School students (8th, 10th, and 12th grades)	USA	✧ For physically active students, frequent use of social media was associated with a greater likelihood of performing daily exercise, and for those who were sedentary, it reduced their activity. ✧ Students who were moderately active and used social media once or twice per month had the highest likelihood of performing vigorous daily exercise.	[24]
***Body Image Concerns and Eating Disorders***			
A systematic review and meta-analysis of 83 articles published between 2008 and 2024	Various	✧ The results showed that high online social media comparison was associated with (i) greater body image concerns, (ii) lower positive body image, and (iii) greater levels of eating disorders.	[53]
A systematic review and meta-analysis of 45 articles published between 2005 and mid-2024 with a focus on adolescent participants	Various	✧ The higher levels of social media engagement were associated with more body image concerns and eating disorder symptoms, with a stronger association with Instagram use and female participants.	[54]
***Other Physical Health Issues***			
Collected from 462 university students	Japan	✧ Physical symptoms should not be regarded solely as outcomes of social media use, but also as important mechanisms through which social media use relates to mental health.	[23]

**Table 3:** Key Findings of the Relationship between Social Media Use and Social Health Issues.

Data Source	Country	Findings	References
***Social Support***			
A summary of research findings between 2007 and 2017 from various databases	Various	✧ Facebook could potentially serve as a source of emotional, informational, and instrumental support, which enhances feelings of belonging, reduces isolation, and increases perceived social support. Yet, not all Facebook interactions are supportive. Therefore, the effects depend on user motivations, community norms, and the quality of interactions rather than platform use alone.	[2]
Through two rounds of data collection in 2019 (304 participants) and 2020 (584 participants)	Japan	✧ Before the COVID-19 pandemic (i.e., 2019), Twitter usage was associated with the formation of social networks with relatively close others, further related to an increase in social support from these people. ✧ During the COVID-19 pandemic (i.e., 2020), the total social support received from Twitter was associated with an increased level of well-being, whereas satisfaction with social networks via Twitter was associated with a decreased level of well-being.	[34]
***Social Capital***			
A summary of research findings between 2007 and 2014 from various databases	Various	✧ Facebook use was associated with greater bridging social capital. Further, the relationship between Facebook use and social capital depends on users’ motivations, patterns of use, and the quality of their online interactions rather than the time spent on the platform.	[15]
A summary of research findings between 2000 and 2014 from various databases	Various	✧ Online social interactions had both positive and negative influences on social well-being. On the one hand, social media enables users to maintain interpersonal relationships regardless of distance and strengthen their sense of belonging to online communities, thereby strengthening both bonding and bridging social capital. On the other hand, excessive reliance on online interactions has been associated with reduced face-to-face communication, increased feelings of loneliness, and more superficial interpersonal relationships.	[3]
Collected from 305 university students	USA	✧ Different social media platforms served distinct social functions.	[36]
Collected from 409 university students in 2022	Japan	✧ Face-to-face social capital was positively associated with better mental health (higher SWB and lower loneliness) and greater perceived physical health, and facilitated the development of online social capital. ✧ Online social capital was not associated with either mental health or perceived physical health.	[22]

**Table 4:** Key Findings of the Relationship between Social Media Use and Health Misinformation.

Data Source	Country	Findings	References
A commentary	N/A	✧ The WHO announced during the COVID-19 pandemic that our society was actually facing an “infodemic”.	[43]
A general review conducted via information from legal perspectives	N/A	✧ Recommendation algorithms amplify popular or emotionally engaging content, regardless of its accuracy, increasing the likelihood that misinformation reaches large audiences.	[10]
A general review conducted via journalism perspectives	N/A	✧ Through empowerment from social media for the general public to disseminate their opinion, people who stayed in the “echo chambers” and were influenced by the “hostile media effect” would publish their own interpretation of available information, creating more health misinformation on social media.	[44]
Data collected from Twitter	N/A	✧ False news spread significantly faster, farther, and more broadly than truthful news, as such news pieces were perceived as more novel and emotionally engaging, making users more likely to share them with others.	[11]

**Table 5:** Proposed Future Research Agenda on the Relationship between Social Media Use and Health.

Future Research Directions	Suggestions	References
From cross-sectional research to causal explanation	✧ More longitudinal panel studies, cross-lagged analyses, natural experiments, and randomized controlled trials should be developed. These approaches would allow us to understand the directionality of effects and identify the causal pathways linking social media use to health outcomes.	[21]
Understanding individual differences and the impact of social media use on health	✧ As prior research on the Differential Susceptibility to Media Effects Model (DSMM) proposes that media effects depend on users’ dispositional, developmental, and social characteristics, future research should therefore investigate which groups are particularly vulnerable to adverse health consequences.	[12]
More focus on social media content, rather than usage time alone	✧ Recent research suggested that social media content exposure and usage motivations may be more important than duration alone to individuals. Therefore, future studies should move beyond simple screen-time measures and examine what types of content users consume, create, and share.	[29,30,33,61]
More reliance on objective behavior measures	✧ As self-reports are vulnerable to recall bias and may not accurately reflect actual behavior, future research should integrate objective behavioral measures, such as digital trace data, smartphone usage logs, ecological momentary assessment, wearable-device data, and platform-generated analytics, as objective measures. Coupled with self-report survey data, researchers can have a more accurate dataset to understand how specific behaviors influence health outcomes.	[20]
Analyze data using a social media portfolio approach	✧ As recent studies adopting a social media portfolio perspective have demonstrated that combinations of platforms are associated with different mental and physical health outcomes, future research should therefore investigate how multiple-platform use shapes health outcomes, which may provide a more realistic understanding of contemporary social media ecosystems and improve the development of targeted health interventions.	[22,23,62]
Examining mental and physical health together	✧ Future studies should adopt integrated models that simultaneously examine physical and mental health outcomes. Particular attention should be paid to mediating and moderating mechanisms involving sleep quality, physical activity, sedentary behavior, musculoskeletal symptoms, and perceived health status.	[22,23]
Develop effective interventions against health misinformation	✧ Future research should evaluate the long-term effectiveness of these interventions and identify conditions under which they work best. More research is needed to investigate how health literacy, social media literacy, critical thinking skills, and trust in scientific institutions influence individuals’ susceptibility to misinformation. Given the rapid development of artificial intelligence, future studies should also examine how AI-generated content may influence the production and dissemination of health misinformation on social media.	[41,42,45]
Understanding social polarization and social impacts	✧ Future research should investigate how social media ecosystems shape societal health outcomes at both individual and collective levels.	[10,63]
Toward an interdisciplinary research agenda	✧ Future research should integrate perspectives and methods from public health, psychology, communication studies, information science, computer science, medicine, and policy studies to address the complex relationships among social media use, health outcomes, misinformation, and platform governance.	[64,65]

## Data Availability

No new data were created or analyzed in this study. Data sharing is not applicable to this article.
